# Inflammaging and Blood Pressure Profiles in Late Life: The Screening for CKD among Older People across Europe (SCOPE) Study

**DOI:** 10.3390/jcm11247311

**Published:** 2022-12-09

**Authors:** Lisanne Tap, Andrea Corsonello, Mirko Di Rosa, Paolo Fabbietti, Francesc Formiga, Rafael Moreno-González, Johan Ärnlöv, Axel C. Carlsson, Harmke A. Polinder-Bos, Regina E. Roller-Wirnsberger, Gerhard H. Wirnsberger, Tomasz Kostka, Agnieszka Guligowska, Rada Artzi-Medvedik, Ilan Yehoshua, Christian Weingart, Cornel C. Sieber, Pedro Gil, Sara Lainez Martinez, Fabrizia Lattanzio, Francesco U. S. Mattace-Raso

**Affiliations:** 1 Department of Internal Medicine, Section of Geriatric Medicine, Erasmus MC, University Medical Center Rotterdam, 3015 GD Rotterdam, The Netherlands; 2 Italian National Research Center on Aging (INRCA), 60124 Ancona, Italy; 3 Geriatric Unit, Internal Medicine Department, Bellvitge University Hospital-IDIBELL-L’Hospitalet de Llobregat, 08907 Barcelona, Spain; 4 School of Health and Social Studies, Dalarna University, 791 31 Falun, Sweden; 5 Division of Family Medicine and Primary Care, Department of Neurobiology, Care Sciences and Society (NVS), Karolinska Institutet, 171 77 Stockholm, Sweden; 6 Academic Primary Health Care Centre, Stockholm Region, 113 65 Stockholm, Sweden; 7 Department of Internal Medicine, Medical University of Graz, 8036 Graz, Austria; 8 Department of Geriatrics, Healthy Ageing Research Centre, Medical University of Lodz, 90-549 Lodz, Poland; 9 Department of Nursing, The Recanati School for Community Health Professions, Faculty of Health Sciences, Ben-Gurion University of the Negev, Beer Sheva 84105, Israel; 10 Maccabi Healthcare Services, Southern Region, Tel Aviv 69978, Israel; 11 Department of General Internal Medicine and Geriatrics, Krankenhaus Barmherzige Brüder Regensburg and Institute for Biomedicine of Aging, Friedrich-Alexander-Universität Erlangen-Nürnberg, 93049 Regensburg, Germany; 12 Department of Geriatric Medicine, Hospital Clinico San Carlos, 28040 Madrid, Spain

**Keywords:** inflammation, neutrophil-to-lymphocyte ratio, hypertension, blood pressure, vascular aging, older adults

## Abstract

The neutrophil-to-lymphocyte ratio (NLR) is a marker for systemic inflammation. Since inflammation plays a relevant role in vascular aging, the aim of this study was to investigate whether NLR is associated with blood pressure profiles in older adults. This study was performed within the framework of the SCOPE study including 2461 outpatients aged 75 years and over. Mean blood pressure values, namely systolic blood pressure (SBP), diastolic blood pressure (DBP) and pulse pressure (PP) were investigated across tertiles of NLR. Change in blood pressure levels in 2 years of follow-up were compared across categories of baseline NLR. Data of 2397 individuals were used, of which 1854 individuals had hypertension. Mean values of blood pressure did not differ across categories of baseline NLR in individuals without hypertension. Individuals with hypertension with a high-range NLR had lower SBP and PP when compared to those in low-range NLR (mean difference SBP −2.94 mmHg, *p* = 0.032 and PP −2.55 mmHg, *p* = 0.030). Mean change in blood pressure in 2 years did only slightly differ in non-clinically relevant ranges, when compared across tertiles of baseline NLR. NLR as a marker of inflammaging was not associated with unfavorable blood pressure profiles in older individuals with or without hypertension.

## 1. Introduction

Hypertension is very common in older adults and viewed as an accelerated form of vascular aging [1,2]. Vascular aging is characterized by breaks in elastic fibers and accumulation of collagen in the arterial wall, resulting in a decline of elastic properties and thus an increase in arterial stiffness [3]. Increased arterial stiffness is also associated with subsequent development of hypertension and related blood pressure alterations, such as a decline in diastolic blood pressure (DBP) and an increase in systolic blood pressure (SBP) and pulse pressure (PP) [4]. Besides age and cardiovascular risk factors, systemic inflammation also plays a relevant role in the rate of vascular aging [5]. However, whether ‘inflammaging’ is associated with vascular aging at older age is not completely clear.

Immunosenescence refers to the significant changes of the immune system with aging [6]. It results in remodeling of specific cell types, higher levels of pro-inflammatory cytokines and seems to induce a permanent low-grade state of chronic inflammation [7]. A variety of biochemical and hematological markers can be measured to assess this systemic inflammation [8]. For instance, the role of C-reactive protein has been widely observed in observational studies in several chronic conditions [9,10,11]. Interestingly, more recent literature indicates that the ratio of blood cells subtypes have a significant prognostic value for cardiovascular diseases [12,13]. The neutrophil-to-lymphocyte ratio (NLR), derived directly from the differential white blood cell count, is a relatively novel marker reflecting the balance between two aspects of the immune system: acute and chronic inflammation (neutrophil count) and adaptive immunity (lymphocyte count) [14]. During (chronic) illness and various pathological states, this balance shifts due to systemic inflammation and oxidative stress. The NLR has proven its prognostic value in several diseases, such as cardiovascular diseases [15,16], infections [17] and several types of cancer [18], in which higher NLR values represent higher rates of inflammation. NLR is an inexpensive, ‘easy to obtain’ and highly available measurement, making it a very accessible tool in clinical practice.

The aim of this study was to explore whether NLR as marker of inflammation is associated with blood pressure profiles in older adults aged 75 years and over with and without hypertension.

## 2. Materials and Methods

The present study was performed within the framework of the Screening for Chronic Kidney Disease among Older People across Europe (SCOPE) study. The SCOPE study (European Grant Agreement no. 436849), is a multicenter 2-year prospective cohort study involving patients older than 75 years attending outpatient services in participating institutions in Austria, Germany, Israel, Italy, the Netherlands, Poland and Spain. Methods of the SCOPE study have been extensively described elsewhere [19]. Participants were requested to sign a written informed consent before entering the study. The study protocol was approved by ethics committees at all participating institutions, and complies with the Declaration of Helsinki and Good Clinical Practice Guidelines.

### 2.1. Inflammation

Blood samples were obtained during baseline visit to assess clinical laboratory tests including the differentiated white blood cell count. The NLR was calculated by dividing the absolute neutrophil count by the absolute lymphocyte count. No specific normal values or cut-off point of NLR in older adults exist and most researchers have explored NLR in categories in their own population. The authors decide to categorize participants in groups of low-range, mid-range and high range values of NLR using tertiles stratified for the presence of hypertension.

### 2.2. Blood Pressure Profiles

Blood pressure measurements were conducted during baseline visit and also every following visit after 1 and 2 years of follow-up using an oscillometric device with a brachial cuff in resting position. SBP and DBP were measured and documented in millimetres of mercury (mmHg). PP, also expressed in mmHg, was calculated as the difference between SBP and DBP. PP is a marker of age-related vascular stiffness in which higher values of PP indicate greater stiffness (i.e., less elasticity) [20]. Since blood pressure was also measured during follow-up, change in blood pressure values (SBP, DBP and PP) was calculated and documented as change in mmHg. Hypertension was registered when present in medical history and/or when antihypertensive medication was used for this indication.

### 2.3. Other Variables

Demographic data and socioeconomic status were documented. Information on alcohol use, smoking status, medical history and use of medication was collected, including the use and type of antihypertensive medication. Additionally, the cumulative illness rating scale for geriatrics (CIRS-G) was calculated [21]. During the study visit, a comprehensive geriatric assessment (CGA) was performed including information on other domains, such as information on cognition and functional status [22].

### 2.4. Statistical Analysis

**Figure 1:**
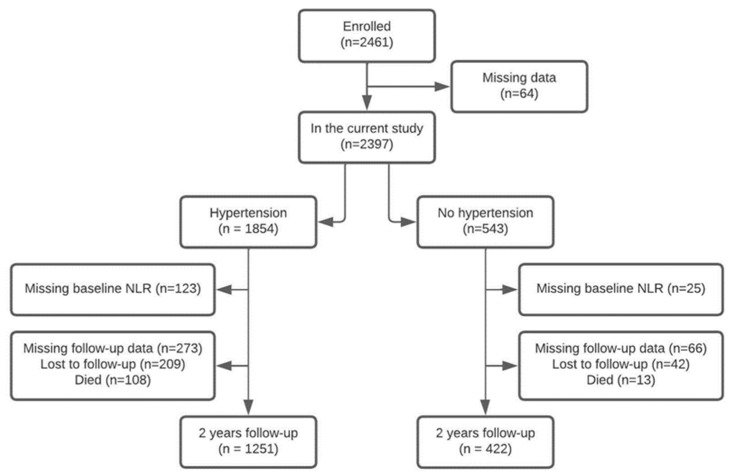
Flowchart on in- and exclusion of individuals within the current study.

Descriptive statistics were expressed as percentage for categorical variables and mean and standard deviation (SD) or median and interquartile ranges (IQR) for continuous variables, depending on normal or non-normal distribution. First, characteristics were compared between participants with and without hypertension using the Chi square test for categorical variables and T-test or Mann–Whitney U test for continuous variables, depending on normal or non-normal distribution. Second, cross-sectional analyses were conducted in which mean values of SBP, DBP and PP were compared across tertiles of NLR using analysis of variance (ANOVA) stratified for the presence of hypertension. Multivariate analyses were also conducted with adjustment for covariates age, sex, BMI, diabetes mellitus, CIRS-G and the use of antihypertensive medication. Third, mean change of SBP, DBP and PP in two years of follow-up were compared across tertiles of NLR stratified for the presence of hypertension using ANOVA. In multivariate analyses, these tests were adjusted for baseline value of SBP, DBP or PP and previous identified covariates. Individuals who were lost to follow-up, died or with missing data were excluded from related analyses (Figure 1). A *p*-value of <0.05 was considered statistically significant.

## 3. Results

In total, 2461 participants were enrolled in the SCOPE study and 2397 participants were included in the analyses as result of missing data on 64 participants. The complete flowchart on the included and excluded individuals is shown in Figure 1. A total of 1854 individuals (77.3%) had a medical history of hypertension and/or used anti-hypertensive medication for this indication and 543 participants (22.7%) had no hypertension. Overall baseline characteristics stratified for the presence of hypertension are presented in Table 1.

Individuals with hypertension were older (80 vs. 79 years, *p* < 0.001), had higher BMI (27.7 vs. 25.8 kg/m^2^, *p* < 0.001), SBP (140 vs. 132 mmHg, *p* < 0.001) and PP (62 vs. 55 mmHg, *p* = 0.001) than individuals without hypertension. Additionally, the prevalence of comorbidities was higher in individuals with hypertension, than in those without hypertension, such as the prevalence of chronic kidney disease (70.4% vs. 50.5%, *p* < 0.001), stroke (6.8% vs. 2.4%, *p* < 0.001) and diabetes mellitus (29.2% vs. 11.8%, *p* < 0.001), resulting in higher comorbidity score (CIRS-G 9 vs. 5, *p* < 0.001). The amount of neutrophils was higher in individuals with hypertension than in those without hypertension (4.0 vs. 3.5 × 10^9^/L, *p* < 0.001), whereas lymphocytes count did not differ between groups (1.7 vs. 1.6 × 10^9^/L), resulting in higher values of NLR in individuals with hypertension (2.4 vs. 2.1, *p* < 0.001). Individuals with hypertension more often used anti-hypertensive medication than individuals without hypertension (94.9% vs. 24.6%, *p* < 0.001). Most frequently, individuals with hypertension used ACE-inhibitors/ARBs (72.5%). They also used beta-blockers (49.4%), diuretics (46.7%) or calcium channel blockers (32.7%).

### 3.1. Individuals with Hypertension

**Figure 2:**
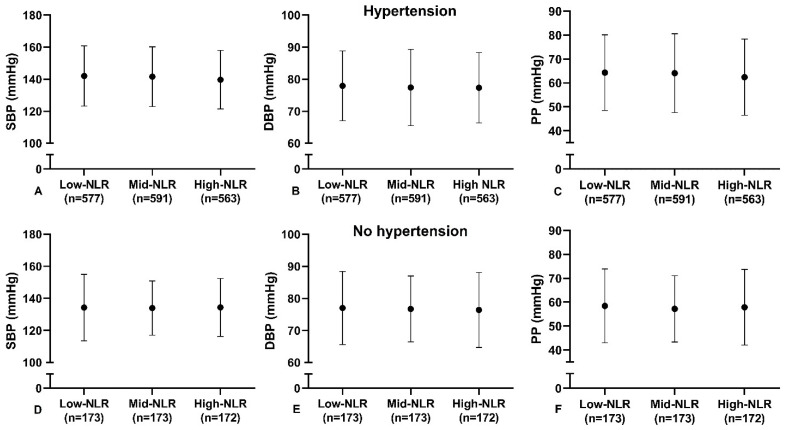
Mean values of blood pressure across tertiles of NLR in individuals with hypertension (**A**–**C**) and no hypertension (**D**–**F**). Dots represent mean values, bars represent standard deviation. Figure **A** and **D**, systolic blood pressure (SBP) in mmHg; Figure **B** and **E**, diastolic blood pressure (DBP) in mmHg; Figure **C** and **F** pulse pressure (PP) in mmHg.

The low-range NLR tertile contained individuals with a value up to 1.95 and the high-range NLR started from 3.01. Mean values of blood pressure across tertiles of NLR are presented in Figure 2A–C.

Mean values of SBP were 142.0 ± 18.8 mmHg, 141.5 ± 18.7 mmHg and 139.7 ± 18.3 mmHg from lowest to highest tertile of NLR, respectively. Individuals in the high-range NLR had slightly lower SBP when compared to low-range NLR (mean difference −2.94 mmHg, 95% CI −5.70; −0.18 mmHg, *p* = 0.032). Mean values of DBP were 77.9 ± 10.9 mmHg, 77.4 ± 11.9 mmHg and 77.3 ± 11.0 mmHg, respectively, with no differences across categories. Mean values of PP were 64.3 ± 15.9 mmHg, 64.1 ± 16.5 mmHg and 62.4 ± 16.0 mmHg, respectively. Individuals in the high-range NLR had lower PP when compared to low-range NLR (mean difference −2.55 mmHg, 95% CI −4.93; −0.18 mmHg, *p* = 0.030).

Mean change of blood pressure levels were compared across tertiles of baseline NLR and presented in Table 2.

Mean changes from lowest to highest tertile of baseline NLR were 2.8 ± 20.8 mmHg, 0.7 ± 21.3 mmHg and 1.4 ± 21.4 mmHg for SBP, 0.4 ± 33.8 mmHg, 0.4 ± 12.2 mmHg and 0.6 ± 12.3 mmHg for DBP and 0.9 ± 17.4 mmHg, 1.1 ± 18.7 mmHg and 0.9 ± 18.2 mmHg for PP (respectively).

Mean change in blood pressure values in 2 years did only slightly differ in non-clinically relevant ranges.

### 3.2. Individuals without Hypertension

The low-range NLR tertile contained individuals with a value up to 1.77, whereas the high-range NLR started from 2.53. Mean values of blood pressure across tertiles of NLR are presented in Figure 2D–F. Mean values of SBP were 134.2 ± 20.8 mmHg, 133.9 ± 16.9 mmHg and 134.3 ± 18.1 mmHg from lowest to highest tertile of NLR, respectively. Mean values of DBP were 77.0 ± 11.4 mmHg, 76.7 ± 10.3 mmHg and 76.4 ± 11.7 mmHg from lowest to highest tertile of NLR, respectively. Mean values of PP were 58.4 ± 15.5 mmHg, 57.2 ± 13.9 mmHg and 57.9 ± 15.9 mmHg from lowest to highest tertile of NLR, respectively. There were no differences in blood pressure levels across categories.

Mean change of blood pressure levels in individuals without hypertension are also presented in Table 2. Mean changes from lowest to highest tertile of baseline NLR were 0.6 ± 19.0 mmHg, 0.6 ± 19.3 mmHg and 2.9 ± 21.1 mmHg for SBP, 1.3 ± 11.9 mmHg, 1.1 ± 11.7 mmHg and 1.7 ± 11.8 mmHg for DBP and 0.1 ± 15.8 mmHg, 1.8 ± 14.4 mmHg and 1.2 ± 17.4 mmHg for PP (respectively). There was no difference in changes of blood pressure levels across categories.

## 4. Discussion

NLR as a marker of inflammaging, was not associated with unfavorable blood pressure profiles in individuals with and without hypertension aged 75 years and over. Mean changes in blood pressure levels during a follow up of 2 years were close to zero and no differences could be observed in changes of blood pressure levels, when comparing individuals with low-range, mid-range or high-range NLR.

The role of oxidative stress and inflammation in vascular aging and development of hypertension is well established [5]. During aging of the immune system, specific cell types will remodel and a permanent state of chronic inflammation is induced [23]. Several studies found that higher levels of C-reactive protein and pro-inflammatory cytokines are associated with increased arterial stiffness [24,25]. Chronic inflammation can affect blood vessel structures and this effect is most likely due to the role of inflammatory state in endothelial dysfunction, by inhibiting endothelium-dependent vasodilatation. In middle-aged adults, a cross-sectional study investigated the possible association between NLR and 24 h blood pressure measurements in patients with newly diagnosed hypertension [26]. Results showed that patients with no antihypertensive therapy in the upper two quartiles of NLR had the highest systolic and diastolic blood pressure. Additionally, a significant association between NLR and high blood pressure load was found. Interestingly, the quartiles of NLR were comparable with the tertiles used in our study, namely first < 1.55, second 1.55–1.92, third 1.92–2.48 and fourth > 2.48. Since this study involves a specific group of untreated patients with hypertension with a mean age of 49 years, the main results could not be compared to our older adults, who commonly have a history of decades with hypertension. Another cross-sectional study performed in England investigated the relation between NLR and 24 h blood pressure measurements in 508 individuals between the age of 18–80 years who underwent the blood pressure measurement for the diagnosis or evaluation of hypertension [27]. A positive association between NLR and arterial stiffness was found. Moreover, increasing NLR was an independent predictor of cardiovascular outcomes. Included patients had a mean age of 58.8 ± 14.0 and this study did not focus on blood pressure profiles and NLR, and therefore these results in younger patients could not be completely compared to our findings.

As far as we are concerned, no studies have investigated the possible association between NLR and blood pressure profiles in old age. We had hypothesized that a higher rate of inflammation would be associated with unfavorable blood pressure levels, however, the findings could not confirm our hypothesis. We took into account also the potential of anti-inflammatory effects of the different type of antihypertensive medication on this association [28,29,30], however adjusting for this potential confounder did not change our results. A potential explanation for our findings could be due to a ceiling effect. There is an association between inflammation and blood pressure levels at younger age, however, in our population of individuals aged 75 years and over, a ceiling effect might explain our findings. Additionally, it is possible that seriously ill patients with unfavorable inflammation and blood pressure profiles or the highest rate of comorbidities, were not included in this study, due to death or no willingness to participate due to health-related issues. Additionally, death during follow-up could have affected our results. Eventually, the annual change in blood pressure levels in our population might be too small to be investigated during a follow-up of 2 years. A study conducted in the USA which included older adults of 75 years and over found a mean annual change in SBP of <1 mmHg in men and circa 2 mmHg in women and a mean annual change in DBP of circa 1.2 mmHg in both sexes [31]. We found quite an opposite result as than what we expected in individuals with hypertension, namely the group with the highest NLR had the lowest SBP and PP. A possible explanation for this finding could lay in the fact that theoretically, patients with hypertension and high NLR represent a group with the least favorable health status. Consequently, those older adults might be the ones with most frequent hospital visits and better health-care surveillance leading to better blood pressure control.

Some limitations of the present study deserve consideration. First, we decided to analyse blood pressure levels across tertiles of NLR, instead of a continuous number which could have affected the results. This decision was based on the fact that no normal values for older adults exist and since it is not known whether or not every step of higher or lower NLR is as relevant. Second, blood pressure values were documented from one measurement per visit, while 24 h blood pressure measurement could have provided another view on blood pressure profiles. Third, we did not took into account changes in medication during follow-up which could have affected the mean levels of blood pressure of time. Fourth, as stated previously, we cannot exclude that the length of follow-up might be too short to investigate a possible relation between inflammation and blood pressure profiles. Fifth, survival bias could have led to a ceiling effect in which individuals with the most unfavorable profiles were not included or could not be followed up. The present study also has strengths. We have studied a large real-world population of older adults in 7 different countries, with no strict inclusion criteria, therefore our findings may apply for a large population of older adults across Europe. Moreover, all data were obtained systematically in participating centers, which makes these results very reliable. Furthermore, NLR is a novel and interesting marker to investigate, so the present study contributes in this relatively new field of research. Additionally, multiple blood pressure values were included, among which SBP, DBP and PP. Since blood pressure profiles can change with aging those three included measurements were able to reflect different angles of vascular aging.

## 5. Conclusions

In conclusion, in older adults with higher rates of inflammation, we expected to find unfavorable blood pressure profiles reflecting elevated arterial stiffness and higher rate of vascular aging. However, this association was not found. The search for contributing factors to accelerated vascular aging even at higher age is still highly relevant in order to recognize individuals at risk for cardiovascular outcomes and optimize possible treatment strategies.

## Figures and Tables

**Table 1:** Overall sample description in individuals with and without hypertension.

Variable	Hypertension (n = 1854)	No Hypertension (n = 543)	*p*-Value
Age, years	80 [77–83]	79 [77–82]	<0.001
Women	1034 (55.8%)	307 (56.5%)	NS
Living alone	456 (24.6%)	119 (21.9%)	NS
BMI, kg/m^2^	27.7 [25.1–30.8]	25.8 [23.4–28.6]	<0.001
Alcohol consumption	449 (24.2%)	161 (29.7%)	0.009
Current Smoker	73 (3.9%)	30 (5.5%)	NS
Former Smoker	698 (37.6%)	201 (37%)	NS
ADL dependent	475 (25.6%)	99 (18.2%)	0.001
iADL dependent	822 (44.3%)	156 (28.7%)	<0.001
CKD	1306 (70.4%)	274 (50.5%)	<0.001
eGFR-BIS, mL/min	52.9 [41.4–62.0]	59.9 [52.5–66.7]	<0.001
TIA	175 (9.4%)	35 (6.4%)	0.030
Stroke	126 (6.8%)	13 (2.4%)	<0.001
Cancer	328 (17.7%)	87 (16%)	NS
COPD	239 (12.9%)	48 (8.8%)	0.011
CHF	360 (19.4%)	38 (7%)	<0.001
Diabetes Mellitus	541 (29.2%)	64 (11.8%)	<0.001
Atrial fibrillation	319 (17.2%)	44 (8.1%)	<0.001
Vascular disease	259 (14%)	43 (7.9%)	<0.001
SBP, mmHg	140 [130–152]	132 [120–147]	<0.001
DBP, mmHg	79 [70–85]	78 [70–83]	NS
PP, mmHg	62 [52–73]	55 [48–67]	<0.001
CIRS-G, total score	9 [6–12]	5 [3–8]	<0.001
CIRS-G, severity index	1.5 [1.3–1.8]	1.4 [1.0–1.7]	<0.001
Neutrophils, 10^9^/L	4.0 [3.2–5.1]	3.5 [2.8–4.4]	<0.001
Lymphocytes, 10^9^/L	1.7 [1.3–2.1]	1.6 [1.3–2.1]	NS
NLR ^1^	2.4 [1.8–3.3]	2.1 [1.6–2.8]	<0.001
Antihypertensives	1795 (94.9%)	139 (25.6%)	<0.001
Calcium channel blockers	606 (32.7%)	16 (3.0%)	<0.001
ACE-inhibitors/ARB	1344 (72.5%)	50 (9.2%)	<0.001
Diuretics	866 (46.7%)	40 (7.4%)	<0.001
Beta-blockers	915 (49.4%)	92 (16.9%)	<0.001

Values are expressed as number (percentage) or median [IQR]. *p*-values are based on chi-squared test for categorical variables and Mann–Whitney U test for continuous variables; ^1^ Data available on 1731 individuals with hypertension and 518 without hypertension. Abbreviations: BMI, Body Mass Index; (i) ADL, (instrumental) Activities of Daily Living; CKD; chronic kidney disease, eGFR-BIS, estimated Glomerular Filtration Rate-Berlin Initiative Study; TIA, transient ischemic attack; COPD, chronic obstructive pulmonary disease; CHF, chronic heart failure; SBP, systolic blood pressure; DBP, diastolic blood pressure; PP, Pulse Pressure; CIRS-G, Cumulative Illness Rating Scale for Geriatrics; NLR; neutrophils-to-lymphocyte ratio; ACE, angiotensin-converting enzyme; ARBs, angiotensin receptor blockers.

**Table 2:** Mean change of blood pressure values across tertiles of NLR in individuals with and without hypertension.

Hypertension (n = 1251)	Low NLR (n = 432)	Mid NLR (n = 429)	High NLR (n = 390)
ΔSBP, mmHg	2.8 ± 20.8	0.7 ± 21.3	1.4 ± 21.4
ΔDBP, mmHg	0.4 ± 33.8	0.4 ± 12.2	0.6 ± 12.3
ΔPP, mmHg	0.9 ± 17.4	1.1 ± 18.7	0.9 ± 18.2
**No Hypertension** **(n = 422)**	**Low NLR** **(n = 152)**	**Low NLR** **(n = 145)**	**Low NLR** **(n = 125)**
ΔSBP, mmHg	0.6 ± 19.0	0.6 ± 19.3	2.9 ± 21.1
ΔDBP, mmHg	1.3 ± 11.9	1.1 ± 11.7	1.7 ± 11.8
ΔPP, mmHg	0.1 ± 15.8	1.8 ± 14.4	1.2 ± 17.4

Values are expressed as mean (±SD); Δ = delta/change; *p*-values are based on one-way ANOVA comparison. Abbreviations: SBP, systolic blood pressure; DBP, diastolic blood pressure; NLR; neutrophil-to-lymphocyte ratio.

## Data Availability

Data will be available for SCOPE consortium on request from the principal investigator, Fabrizia Lattanzio, Italian National Research Center on Aging (IRCCS INRCA), Ancona, Fermo and Cosenza, Italy. f.lattanzio@inrca.it.
